# Catalysis of the Thermal Decomposition of Transition Metal Nitrate Hydrates by Poly(vinylidene difluoride)

**DOI:** 10.3390/polym13183112

**Published:** 2021-09-15

**Authors:** Lasanthi Sumathirathne, William B. Euler

**Affiliations:** Department of Chemistry, University of Rhode Island, 140 Flagg Road, Kingston, RI 02881, USA; lasanthis@uri.edu

**Keywords:** PVDF, nitrate thermal decomposition, catalysis

## Abstract

Poly(vinylidene difluoride) (PVDF) doped with transition metal nitrate hydrates are cast into thin films giving a high β-phase content. Analysis of the thermal behavior of the doped PVDF shows that the decomposition of the metal (II) nitrate hydrates to metal (II) oxides is catalyzed by the PVDF, as evidenced by reduction in the decomposition temperature by as much as 170 °C compared to the pure metal salts. In contrast, there is little to no apparent catalysis for the decomposition of the metal (III) nitrate hydrates. The FTIR spectra of the gas phase decomposition products show H_2_O and NO_2_ are the major components for both PVDF-doped material and the pure metal nitrate hydrates. A mechanism for the role of PVDF is proposed that uses the internal electric field of the ferroelectric phase to orient the nitrate ions and polarize the N–O bonds.

## 1. Introduction

Poly(vinylidene difluoride) is a well-studied semi crystalline fluoropolymer that, depending upon the phase, can be nonpolar, polar, pyroelectric, piezoelectric, or ferroelectric [1,2,3,4,5,6,7,8,9]. The non-polar α-phase has monomer units that alternate between *s-trans* and *s-gauche* conformations, denoted as TGTG′ (the prime indicates a dihedral angle with the opposite sign); the polar β-phase has the all *s-trans*, TTTT, structure; while the weakly polar gamma phase has a TTTGTTTG’ monomer sequence [1,2,3,4,5,6].

The ferroelectric β-phase is generally the most sought after due to its electroactivity [7,8,9]. Common PVDF preparation methods give a high amount of non-polar α-phase. The usual methods to increase the amount of β-phase are by electrically poling a stretched polymer below the melting point [1,6], preparing thin films with polar solvents [7], or synthetically by co-polymerizing 1,2-difluoroethylene with 1,1,2-trifluoroethylene or tetrafluoroethylene [6,8]. Some of the above-mentioned methods will give a high fraction of γ-phase, as well [6].

It has been reported for several years that addition of dopants to PVDF leads to deposition of films with significantly modulated electromagnetic properties [3,5,7,10,11,12,13,14,15,16]. For example, when PVDF is doped with Co(II) ions, the metal ions weakly bond with fluorine atoms of PVDF, which leads to a cooperative effect aligning the bond dipole moments of the polymer chain that leads to a higher fraction of β-phase [13]. Thin films prepared by doping with Ca(II) [4] and Zn(II) [15] also gave a significantly higher fraction of β-phase due to the charge effect. In addition, doping PVDF significantly modulates the fluorescent properties of surface fluorophores, which are used in sensors [17]. Thus, we wished to examine the effects of metal oxides doped into PVDF. Further, a recent report demonstrated that the thermal decomposition temperature of PVDF thin films doped with Zn(NO_3_)_2_·6H_2_O to form ZnO was significantly lower than found in the pure zinc nitrate hydrate [17]. This observation prompted us to investigate the role of PVDF in the thermal decomposition of a series of metal nitrate hydrates doped into the polymer.

We prepared high β-phase fraction PVDF doped thin films doped with six transition metal ions: Zn^2+^, Cu^2+^, Ni^2+^, Co^2+^, Fe^3+^, and Cr^3+^ using the corresponding hydrated metal nitrates. Thermal gravimetric analysis (TGA) was used to determine the decomposition temperature of the transition metal salts for both pure metal nitrates and PVDF-doped materials. The solid phase products were characterized by infrared spectroscopy (FTIR) and powder x-ray diffraction (XRD), which showed that the intermediates in thermal decomposition included metal hydroxide nitrate double salts and the final products were metal oxides. The gas phase products were examined concurrently with the TGA experiment using FTIR, where the primary products were NO_2_ and H_2_O in all cases. A mechanism invoking the local polarity of the PVDF is proposed, which accounts for the experimental observations. The catalysis by the PVDF is effective when the products are M(II) oxides but much less so when the products are M_x_O_y_ (x ≠ y ≠ 1).

## 2. Experimental

PVDF (M_W_ ~534,000 g·mol^−1^), Zn(NO_3_)_2_·6H_2_O, Cu(NO_3_)_2_·2.5H_2_O, Ni(NO_3_)_2_·6H_2_O, Co(NO_3_)_2_·6H_2_O, Fe(NO_3_)_3_·9H_2_O, and Cr(NO_3_)_3_·9H_2_O, were purchased from Sigma Aldrich. Analytical grade N,N-dimethylformamide (DMF) and acetone were purchased from Fisher Scientific and used without any further purification.

Glass slides were used as the substrate to deposit the polymer layer using dip coating. Before use, all the glass slides were cleaned by sonicating for 15 min in ethanol, washed 3 times with distilled water, and finally sonicated again for 15 min with distilled water. The cleaned glass slides were dried with N_2_ gas. A solvent mixture was prepared by adding acetone to DMF in a 9:1 (*v*/*v*) ratio and the PVDF solution was a 10% (*w*/*v*) composition in the prepared solvent mixture. The polymer mixture was sonicated with a Branson 3510 sonicator for 3 h at 40 °C in a closed glass vial until all the PVDF powder was dissolved in the solvent. To prepare composite films, the amount of transition metal salt required to make a 4 mol% metal ion in the PVDF was added to the prepared polymer solution and sonicated for another 2 min without heat. Polymer films were prepared in an N_2_ environment (to maintain the relative humidity below 20%) using an MTI model TL0.01 dip-coater. Twenty mL of polymer solution was placed in a 25.0 mL beaker and the cleaned glass slide was inserted into the polymer solution and withdrawn at 124 mm/min pull rate to make a uniform film. After dip coating, the thin film was annealed in a 60 °C oven for 2 min.

The TGA (TA instruments, New Castle, DE, USA’ Q5000) combined with the FTIR (Thermo scientific Nicolet 6700, Waltham, MA, USA) was used to simultaneously measure the mass loss of the solids and the FTIR spectra of the gas phase products. The aluminum pans were packed with ~20.5 mg of samples and then analyzed from 25 to 400 °C, with a 5 °C/min heating rate under 20 mL·min^−1^ N_2_ flow. The temperature of both the gas transfer tube and the gas FTIR cell were kept at 150 °C. The lag time between the evolution of the gas phase products at the TGA pan and the IR detection was about 2 min.

Solid products heated to different temperatures were prepared in a MTI Corporation EQ-CVF-1300T furnace under an N_2_ atmosphere with flow of 20 mL·min^−1^. The sample was ramped to the final temperature at 5 °C·min^−1^ and then annealed at the target temperature for 60 min. Then, the samples were cooled to room temperature for XRD and FTIR measurements. FTIR spectra of the solid products were measured at room temperature between 650 and 4000 cm^−1^ using a Perkin-Elmer Spectrum 100 instrument in the ATR mode with 4 cm^−1^ resolution. The XRD measurements were done with a Rigaku Miniflex 600 diffractometer using a Cu Kα radiation source with 0.154 nm wavelength, an accelerating voltage of 40 KV, and 15 mA tube current. All the powder samples of pure metal nitrate hydrates were analyzed with 0.01 degrees step size and speed of 8.00 degrees·min^−1^ with a 5 mm diameter cavity sample holder with a zero-diffraction silicon background. For polymer samples, several thin film samples were stacked on each other and attached to a ring shape XRD sample holder using carbon tape with 0.01 degrees step size and speed of 0.25 degrees·min^−1^. All the diffracted data were compared with the International Center for Diffraction Data (ICDD) reference data library to identify the crystalline products.

## 3. Results and Discussion

**Figure 1:**
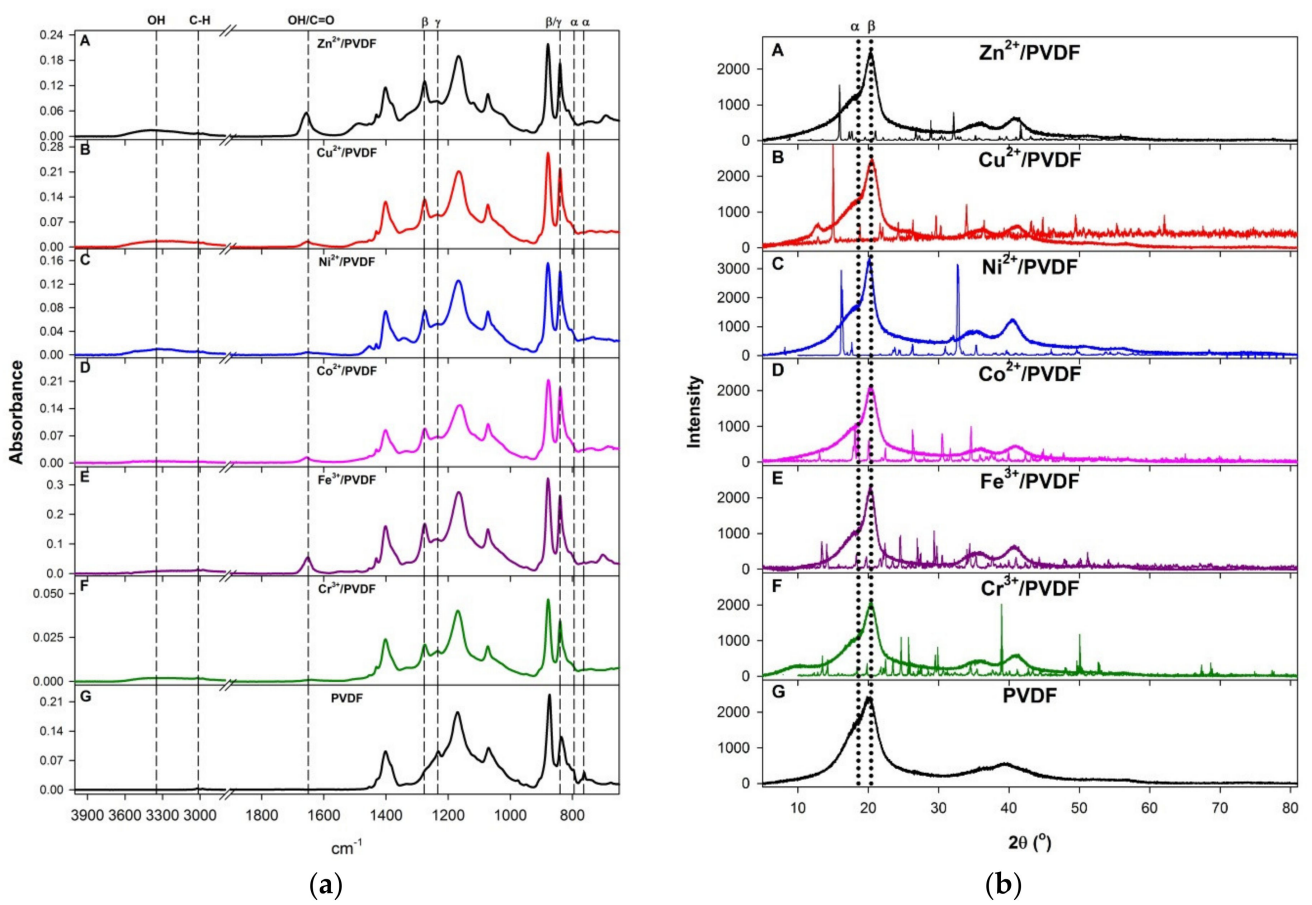
(**a**) FTIR spectra of solid state PVDF-doped/undoped material at room temperature. (**b**) XRD data for PVDF thin films (broad lines) and pure metal nitrate hydrates (sharp lines) at room temperature. A: Zn^2+^/PVDF. B: Cu^2+^/PVDF. C: Ni^2+^/PVDF. D: Co^2+^/PVDF. E: Fe^3+^/PVDF. F: Cr^3+^/PVDF. G: PVDF.

PVDF was doped with six different metal nitrate hydrates: Zn(II), Cu(II), Ni(II), Co(II), Fe(III), and Cr(III). The doping level was set at 4 mol%, which was low enough to prevent aggregation of the dopant but high enough to provide good signal to noise in the measurements made. Figure 1 shows the FTIR spectra and XRD diffractograms of the as prepared films. As shown in Figure 1a, the undoped PVDF thin film is primarily in the α-phase with small amounts of β- and γ-phase, as indicated by the large peak at 764 cm^–1^ (α-phase marker) and the smaller features at 840 (β- and γ-phase marker), 1275 cm^–1^ (β-phase marker), and 1235 cm^–1^ (γ-phase marker) [1,4,6]. Upon doping with metal nitrate hydrates, there were a number of significant changes in the IR spectrum, indicative of changes in the phase composition of the PVDF. There is nearly complete loss of the α-phase and a large increase in the β-phase (and perhaps, the γ-phase). The β-phase composition was calculated using [7,10].

(1) $$F\left(\beta \right)=\frac{A_{840}}{\left(\frac{K_{840}}{K_{764}}\right)A_{764}+A_{840}}$$

Here, *A*_840_ and *A*_764_ are the absorbance values at 840 and 764 cm^−1^, respectively, and *K*_840_ = 7.7 × 10^4^ cm^2^·mol^−1^ and *K*_764_ = 6.1 × 10^4^ cm^2^·mol^−1^ are the absorption coefficient values for those corresponding peaks [4,6]. The nitrate ion has an absorption in this region of the IR spectrum (~805 cm^−1^) so all of the peaks in the 720–850 cm^−1^ range were deconvoluted (Figure S1) to determine the contribution, if any, by nitrate absorption under the 764 and 840 cm^−1^ peaks. While the peak maxima and linewidths for the nitrate absorption shift a little bit for different metal ions (Table S1), in no case is the linewidth of the nitrate absorption large enough to interfere with the peaks at 764 and 840 cm^−1^, supporting the validity of the composition determinations for doped PVDF. As shown in Table 1, doping with metal ions gives a significantly higher β-phase fraction when compared with undoped PVDF. The charge of the metal ion does not appear to make a difference in the β-phase fraction.

In addition, there is retention of some of the DMF solvent and the water of the dopant hydrates. The small and broad peaks centered around 3400 cm^−1^ is assigned to an O–H stretch from occluded H_2_O. There also is a feature in the 1660 cm^−1^ region that consists of multiple peaks that arise from a C=O stretch (from DMF) or an O–H bend (from H_2_O), consistent with the literature [15]. Table S1 gives the deconvoluted peaks in this spectral region. Finally, examination of the C–H stretching peaks of PVDF in the 3000 cm^−1^ region is unchanged between the undoped and all of the doped samples. This indicates that the partially positive hydrogen atoms on the polymer backbone are not involved in any hydrogen bonding [18] with any of the negatively charged species (H_2_O^δ−^, NO_3_^−^, or DMF, which has a negative O^δ−^) in the film. 

X-ray diffraction analysis was also carried out to identify the initial phase composition of PVDF at room temperature, as shown in Figure 1. The crystalline peaks corresponding to pure metal nitrate hydrates are absent when they are doped in PVDF thin films. This indicates that the dopant is fully dissolved in the polymer matrix after deposition from the solvent. All of the XRD data was deconvoluted to determine the effect of doping. In all cases, the diffractograms could be fit with six Gaussian peaks in the range of 5 to 45° 2θ, as shown in Figure S2. In undoped PVDF, these peaks were found at 18.6° (202/020), 35.1° (200), and 39.5° (211), assigned to α-phase crystallites, and 20.3° (200/110), assigned to the β-phase [6,7,14,19,20,21,22,23]. The other two peaks, found at 19.4° and 39.5° were very broad and are assigned to the amorphous background. When PVDF is doped with metal ions, the amount of α-phase features decrease in intensity and the β-phase feature increases. The deconvoluted diffraction peaks are fond at the same angles in the doped and undoped samples, within the error of the measurement, except the α-phase peak at 39.5°, which shifts to 40.8° in all of the metal-ion doped samples.

The XRD data were used to calculate the fraction of β-phase crystallinity using [19].

(2) $$X_{\beta }=\frac{\sum I_{\beta }}{\sum \left(I_{\beta }+I_{\alpha }\right)}$$

Here, *X_β_* is the beta-phase fraction, *I_β_* is the integrated area of *β*-phase peaks, and *I_α_* is the integrated area of the *α*-phase peaks. The calculated values (Table 1) mainly show that the beta fraction increases with metal doping. When these values are compared with FTIR data, it shows there is no trend between the two sets of data.

**Figure 2:**
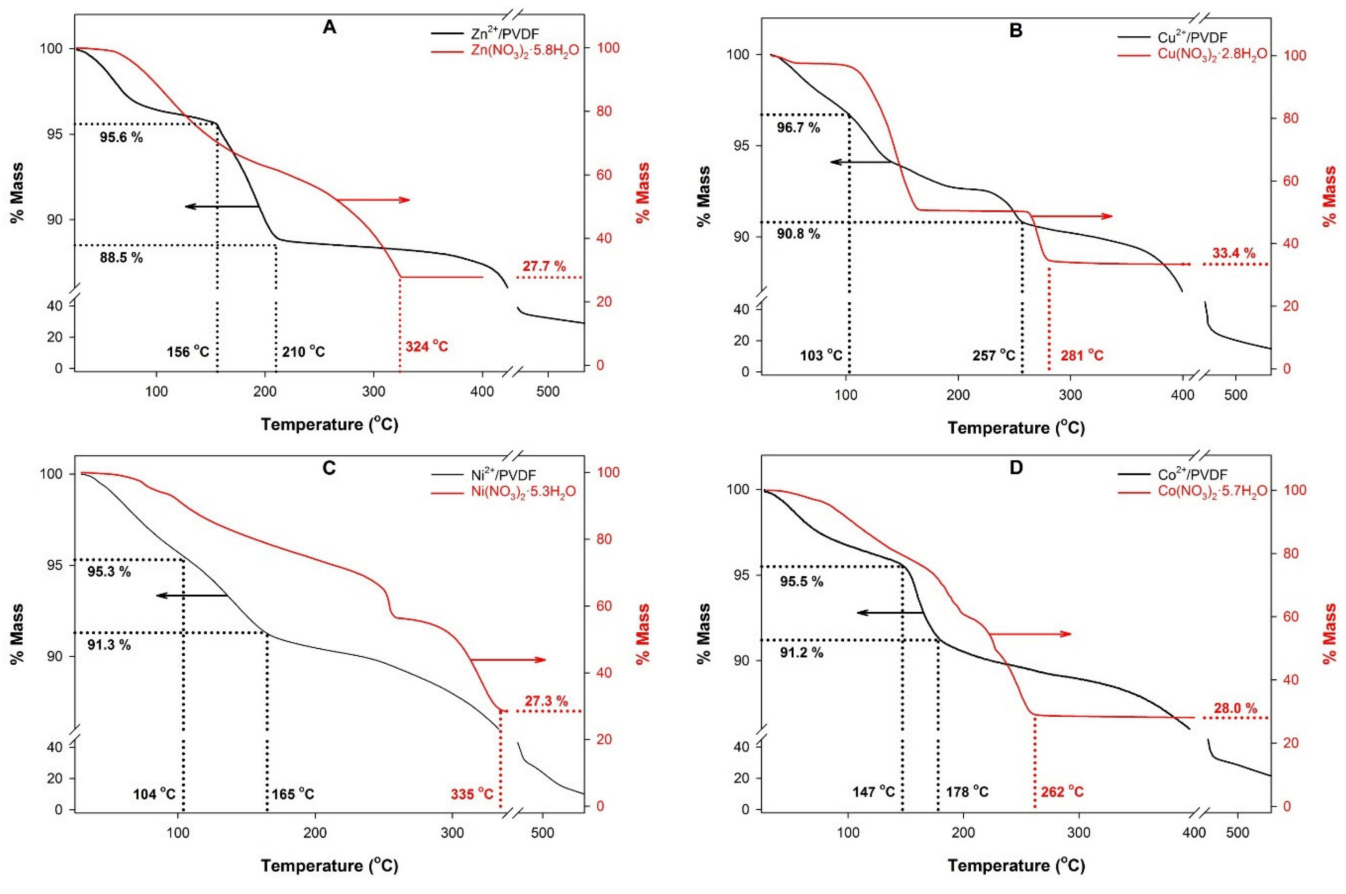

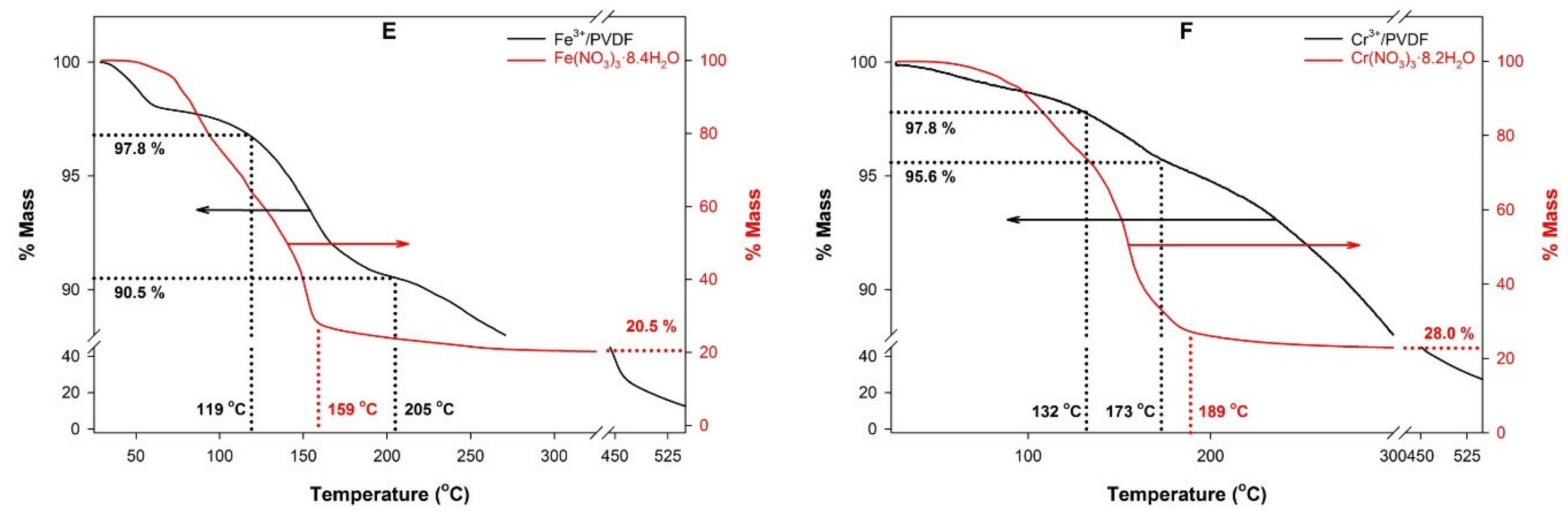
TGA of PVDF-doped materials (black line, left axis) and corresponding pure metal nitrate (dark red line, right axis). The stoichiometries shown for the metal nitrate hydrates were determined by the mass losses. (**A**) Zn^2+^/PVDF and Zn(NO_3_)_2_·5.8H_2_O. (**B**) Cu^2+^/PVDF and Cu(NO_3_)_2_·2.8H_2_O. (**C**) Ni^2+^/PVDF and Ni(NO_3_)_2_·5.3H_2_O. (**D**) Co^2+^/PVDF and Co(NO_3_)_2_·5.7H_2_O. (**E**) Fe^3+^/PVDF and Fe(NO_3_)_3_·8.4H_2_O. (**F**) Cr^3+^/PVDF and Cr(NO_3_)_3_·8.2H_2_O.

The thermal properties (TGA, room temperature to 400 °C) of doped PVDF are shown in Figure 2 (PVDF has no significant mass loss in this range, either doped or undoped). The TGA of pure metal nitrate hydrates are also shown for comparison purposes. When 2+ charged metal ions are doped into PVDF, the shape of the TGA curve is similar to the corresponding pure metal nitrate hydrate data up to 400 °C (where the PVDF begins to decompose), but the onset temperatures of the reactions are reduced, indicating that the activation energy of the decomposition has been reduced and that the PVDF acts as a catalyst. In contrast, this shift is small or at higher temperature for 3+ charged metal nitrates. The lower temperature reactions (water loss, intermediate formation) have less systematic changes. This is in agreement with a previous report for Zn^2+^-doped PVDF, where the decomposition onset temperature was independent of the dopant concentration [15]. The TGA data shows that both pure metal nitrate hydrates and doped PVDF material have two distinguishable mass losses below 400 °C. XRD confirmed that all of the pure metal nitrate hydrates thermally decompose to M_x_O_y_ oxides where x = y = 1 for M = Zn^2+^, Cu^2+^, Ni^2+^, x = 2 and y = 3 [17,24] for M = Fe^3+^ and Cr^3+^ while Co^2+^ gives x = 3 and y = 4 [25,26,27]. Knowing the final product allowed determination of exact H_2_O stoichiometry of the reactants, which was used to confirm the mole fraction of metal ion in the doped films, given in Table 1. Assuming that the decomposition in the doped films leads to the same oxides allows determination of the mole fraction of metal ion in the doped PVDF films, shown in Table 1. The agreement between the metal ion concentration in the casting solution and the doped PVDF films is modest, at best.

Based on the multiple steps observed in the TGA data, the following sequence of reactions for the pure metal hydrates is proposed:

(3) $$M({\mathrm{NO}}_{3}{)}_{z}\cdot {\mathrm{pH}}_{2}O(s)\;\overset{T_{1}}{\to }\;{\left[M({\mathrm{NO}}_{3}{)}_{z}\right]}_{\left(1-\alpha \right)}\cdot [M(\mathrm{OH}{)}_{z}{]}_{\alpha }(s)+z\alpha \;{\mathrm{NO}}_{2}(g)+¼z\;O_{2}(g)+(p-½\alpha z)H_{2}O(g)$$

$$x\;{\left[M({\mathrm{NO}}_{3}{)}_{z}\right]}_{\left(1-\alpha \right)}\cdot [M(\mathrm{OH}{)}_{z}{]}_{\alpha }(s)\;\overset{T_{2}}{\to }\;M_{x}O_{y}(s)+\mathrm{zx}(1-\alpha )\;{\mathrm{NO}}_{2}(g)+\text{¼}[\mathrm{zx}(2-\alpha )-2y]\;O_{2}(g)+\text{½}\mathrm{xz}\alpha \;H_{2}O(g)$$

To confirm the proposed reactivity of pure metal nitrate hydrates, we measured the FTIR spectra of the gases evolved concurrently with the TGA mass losses. In addition, the XRD of the solid residues collected at critical temperatures was measured. Figure S3 shows the volatile decomposition products found from the simultaneous TGA/FTIR analysis. These gas phase products depend on the decomposition temperatures. The main gas phase products observed in all samples were H_2_O (3280 cm^–1^) [28,29] and NO_2_ (1630 cm^–1^) [21,30,31,32,33]. A strong feature assigned to HNO_3_ (875, 1325, and 1720 cm^–1^) [33,34] emission can be observed for Fe(III) and Cu(II) doped samples and a small N_2_O_5_ (1055 cm^–1^) [33,34] emission in the Ni(II) and Cr(III) samples. The emission of HNO_3_ must be a result of the gas phase reaction between NO_2_, O_2_, and H_2_O. The insets in Figure S3 show the gas phase evolution of the H_2_O and NO_2_ peaks. In all cases, the concentration of H_2_O continually increases throughout the entire temperature range. This is consistent with the initial salts losing the water of hydration at lower temperatures, water that may have been formed during reaction, and, at the highest temperatures, water desorbed from the surface of the oxide products. In contrast, NO_2_ increases to a maximum at a salt-dependent temperature and then decreases to zero at higher temperatures. With the exception of Co(NO_3_)_2_·5.7H_2_O, the peak maximum of the NO_2_ evolution agrees with the temperature of the onset of the final plateau, which is the temperature at which the final oxide product is fully formed. The exception for the Co^2+^ salt is likely associated with the observation that some of the cobalt is oxidized to Co^3+^, which is the only oxide where the oxidation state of the metal is different from that of the reactant.

Confirmation of the oxide products of the thermal decomposition of the metal nitrate hydrates was done using XRD, as shown in Figure S4. Bulk samples were heated to 400 °C, well above the final plateau in the TGA for all samples, and held at that temperature for 1 h. After the samples had cooled, the XRD measurements were taken. In all cases except cobalt, the oxide product contained the metal in the same oxidation state as the initial salt (ZnO, CuO, NiO, Fe_2_O_3_, and Cr_2_O_3_). For Co(NO_3_)_2_·5.7H_2_O, the final product is Co_3_O_4_, which is mixed valent (2+, 3+). This is consistent with the fact that the oxidation of CoO by O_2_ to Co_3_O_4_ is thermodynamically favorable (ΔG° (262 °C) = −178 kJ/mol, using data from the NIST web book [35]).

For each metal salt, a sample was collected at an intermediate temperature near the onset of the intermediate product. The XRD measurements for these intermediates are shown in Figure S5. For the zinc, copper, and nickel salts, there was clear evidence of hydroxide/nitrate double salts (Zn_3_(OH)_4_(NO_3_)_2_, Cu_2_(OH)_3_(NO_3_), and Ni_3_(OH)_4_(NO_3_)_2_). The intermediates for the chromium and iron were noncrystalline. Finally, in the case of the cobalt salt, the diffractogram has a few weak peaks that do not fit a double salt, but may arise from the early growth of Co_3_O_4_.

**Figure 3:**
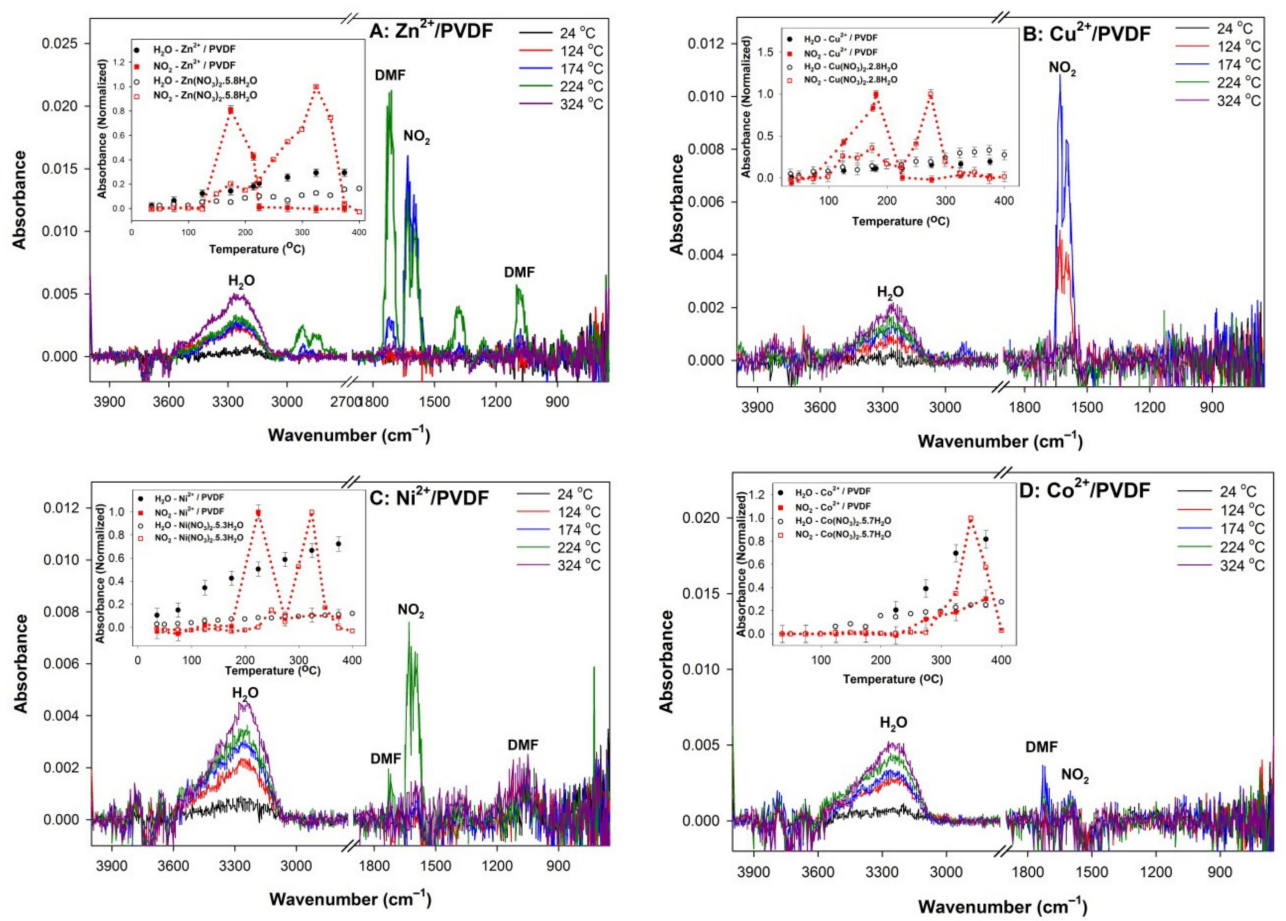

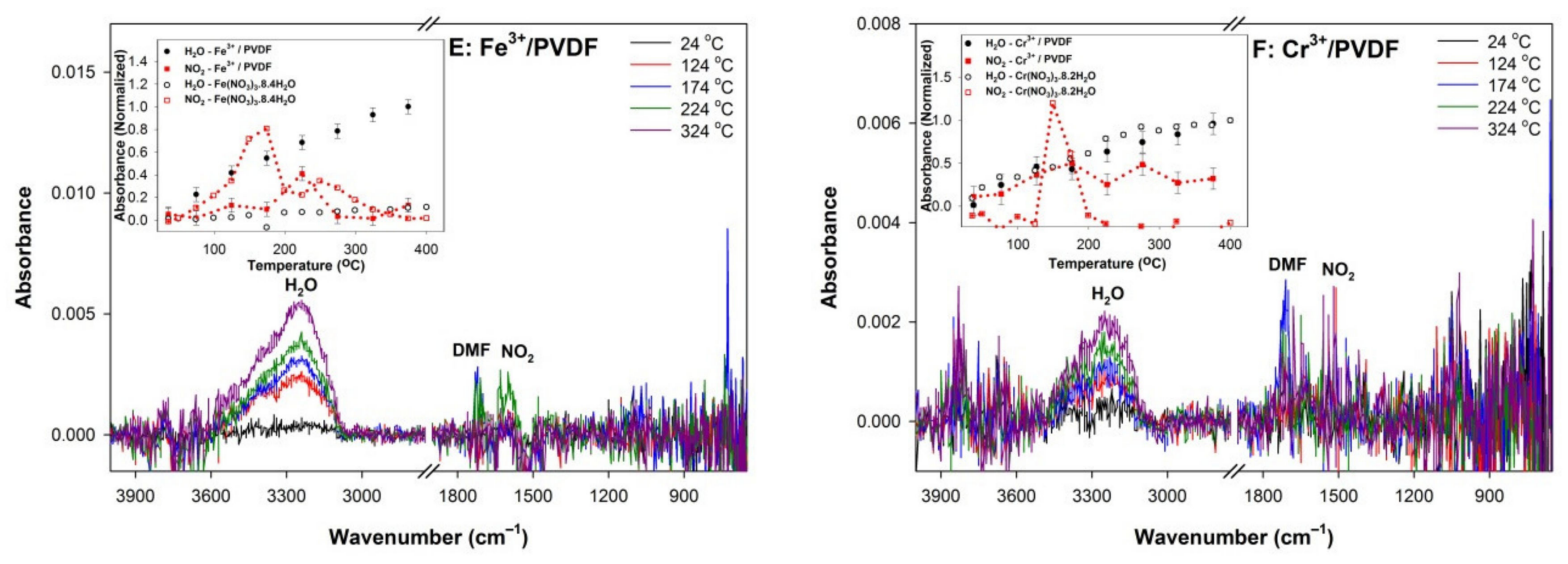
Gas phase IR spectra of the volatile products of the thermal decomposition of PVDF-doped materials. (**A**) Zn^2+^/PVDF; (**B**) Cu^2+^/PVDF; (**C**) Ni^2+^/PVDF; (**D**) Co^2+^/PVDF; (**E**) Fe^3+^/PVDF; (**F**) Cr^3+^/PVDF. The insets show the temperature dependence of the normalized IR peaks for H_2_O (black) and NO_2_ (red) thermal decomposition products. The filled symbols are for metal-doped PVDF and the open circles are for the pure metal nitrate hydrate. The dotted lines on the NO_2_ points are guides for the eye.

When the metal nitrate hydrates are doped into PVDF, the thermal decomposition of the dopants follow the same reactivity as the pure metal salts, but at different temperatures. Figure 3 shows the FTIR spectra of the gas phase thermal decomposition products of the PVDF doped materials. As with the pure metal salt hydrates, the primary emissions are NO_2_ and H_2_O as the temperature is increased. Peaks assigned to loss of DMF (1100, 1400, and 1710 cm^−1^) [36] are observed when PVDF is doped with Zn^2+^ (strong), Ni^2+^ (weak), and Cr^3+^ (weak). The insets in Figure 3 show a comparison of main volatile decomposition products of both PVDF-doped material and pure metal nitrates. The loss of H_2_O and NO_2_ becomes more significant at lower temperatures for the PVDF-doped samples compared to the pure metal nitrate hydrates. The evolution of NO_2_ maximizes at just below the onset temperature of the final oxide formation for the decomposition of the pure metal nitrate hydrates. This trend is also true for the PVDF doped materials, with the observation that for Zn^2+^, Cu^2+^, Ni^2+^, and Co^2+^, this maximum is at significantly lower temperature. The reverse is true for Fe^3+^ and Cr^3+^ doped PVDF. This implies that the PVDF host catalyzes the thermal decomposition of the nitrate ion for the 2+ ions but inhibits this reaction for the 3+ ions.

It is also of note that the amount of NO_2_ evolved is very small for the Co^2+^, Fe^3+^, and Cr^3+^ doped PVDF films. The unique feature of these three dopants is that the final oxide product has the metal in the 3+ oxidation state. It has been previously reported [37] that oxides containing 3+ transition metals catalyze the decomposition of NO_2_ to N_2_ and O_2_ while the 2+ metal oxides are less effective. The same behavior is observed in the PVDF doped materials.

Figures S6 and S7 show the XRD measurements of metal-doped PVDF samples heated to the onset temperatures of the first and second mass losses. As expected, the diffractograms are dominated by the PVDF. For samples heated above 150 °C, the loss of the β-phase is apparent. There are a few additional broad peaks that can be attributed to the same decomposition products as found in the pure metal nitrate hydrates. The FTIR spectra for the same set of samples are shown in Figures S8 and S9. For the samples heated to the intermediate temperature, there is a weak OH band in the 3300 cm^−1^ region consistent with the presence of hydroxide or residual H_2_O. However, the solvent peaks in the 1650 cm^−1^ region are gone or considerably reduced, indicating that the OH stretch should be assigned to metal hydroxides. At the higher temperature, the spectra around 3300 cm^−1^ are baseline, demonstrating the loss of hydroxide and water. Consistent with the XRD results, the FTIR spectra show the loss of β-phase for any samples heated above 150 °C.

Assuming that the thermal decomposition of the metal nitrate hydrates is the same in the presence and absence of PVDF, the proposed reactions are:

$$\{{\left[{\mathrm{CH}}_{2}{\mathrm{CF}}_{2}\right]}_{\left(1-f\right)}[M({\mathrm{NO}}_{3}{)}_{z}\cdot {\mathrm{pH}}_{2}O\cdot \mathrm{qDMF}{]}_{f}{\}}_{n}(s)\;\overset{T_{1}}{\to }$$

{[CH_2_CF_2_]_(1 − f)_[(M(NO_3_)_z_)_(1 − α)_·(M(OH)_z_)_α_]_f_}_n_(*s*) + nfzα NO_2_(*g*) + ¼nfzα O_2_(*g*) + nf(p − ½αz) H_2_O(*g*) + nfq DMF(*g*)

$$\{{\left[{\mathrm{CH}}_{2}{\mathrm{CF}}_{2}\right]}_{\left(1-f\right)}{\left[(M({\mathrm{NO}}_{3}{)}_{z}\right)}_{\left(1-\alpha \right)}\cdot (M(\mathrm{OH}{)}_{z}{)}_{\alpha }{]}_{f}{\}}_{n}(s)\;\overset{T_{2}}{\to }$$

{[CH_2_CF_2_]_(1 − f)_[M_x_O_y_]_f/x_}_n_(*s*) + nfz(1 − α) NO_2_(*g*) + ¼nf[zx(2-α) − 2y/x] O_2_(*g*) + ½nfzxα H_2_O(*g*)

Under the hypothesis that the thermal decomposition reactions of the metal nitrate hydrates is the same independent of the presence of PVDF, the changes critical temperatures T_1_ and T_2_ indicate that the PVDF does influence the reactivity. In the cases of 2+ metal ions, the reduction in the critical temperatures implies that the PVDF acts as a catalyst. In the case of Zn^2+^, as reported previously [15], the thermal decomposition is independent of the metal salt concentration, further supporting the hypothesis that PVDF acts as a catalyst.

**Scheme 1:**
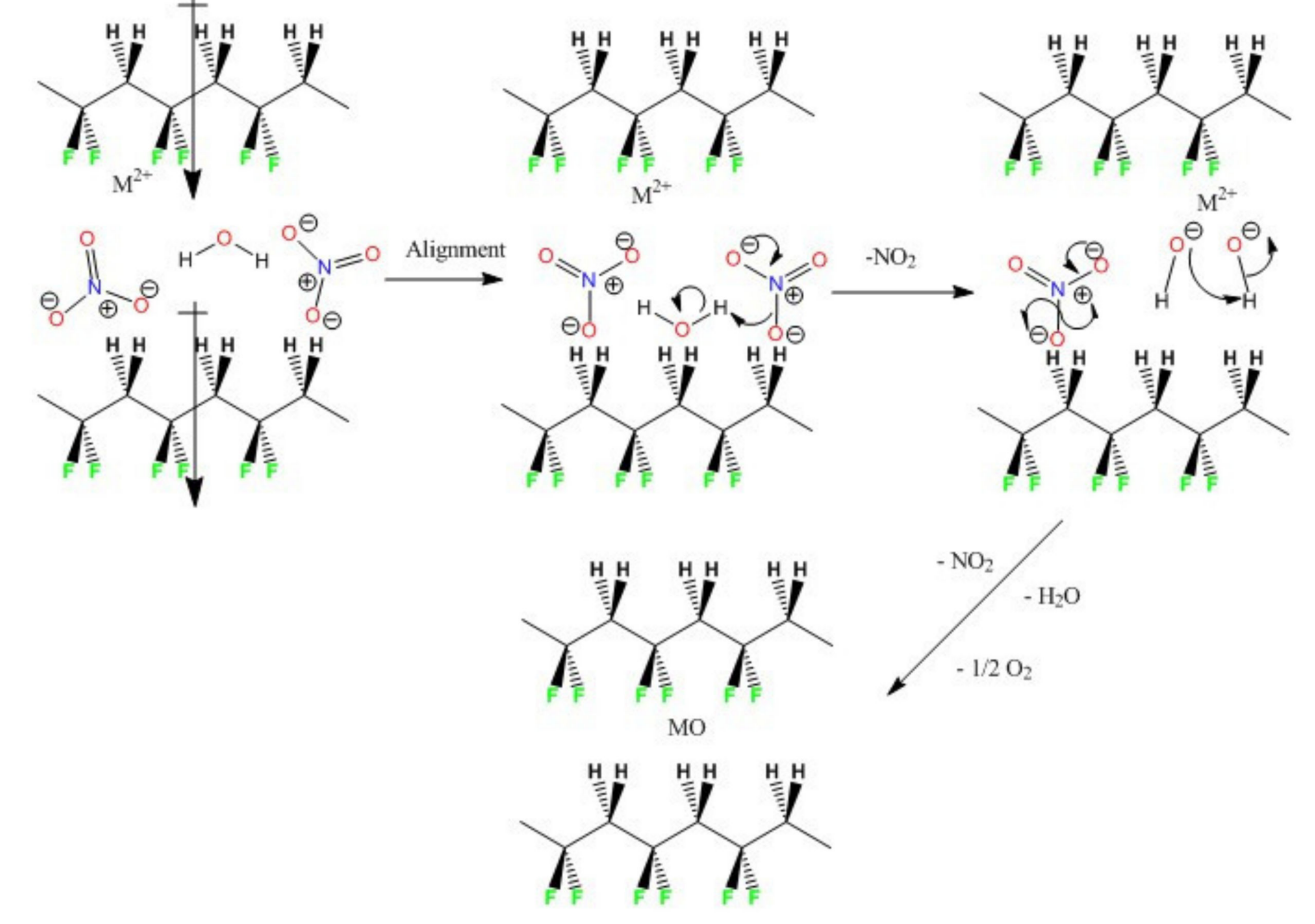
Proposed mechanism for the decomposition of nitrate ion in PVDF.

Scheme 1 shows a possible mechanism for the decomposition of the nitrate ion in the PVDF matrix to form the product oxide. The initially prepared room temperature samples show no crystallinity associated with the dopant metal salt, indicating that the dopant is a solute, dispersed throughout the PVDF matrix. This allows the ions, specifically the nitrate ions, to freely rotate about the C_3_ axis without any restrictions from neighboring ions. Thus, the nitrate ion can align with the local dipole moment and/or the permanent ferroelectric moment of the PVDF (it may be that the alignment of the PVDF into the β-phase is cooperative with the rotation of the nitrate ion). There is no shift in the C–H stretching peaks in the IR spectra, which implies (unsurprisingly) that there is no hydrogen-bonding between the C–H and the weak base NO_3_^−^, but the local electric field may polarize and weaken the N–O bond. Likewise, the partially negative O end of H_2_O can also align with the positive end of the PVDF dipole. In those cases, where the nitrate ion and water are near each other, the N–O bond can cleave heterolytically to form NO_2_ and O^2−^. If the oxide ion finds a metal ion, then an oxide can form. If the O^2−^ encounters an H_2_O, then two OH^−^ ions can form. At elevated temperature the OH^−^ ions are mobile and can form M(OH)_x_ crystallites. The other option is that the local dipolar field induces homolytic cleave of an N–O bond, then the products are NO_2_ and an O· radical, which combines with another O· to form O_2_. At higher temperatures, metal hydroxides can dehydrate to form metal oxide products.

The proposed mechanism is consistent with the observed measurements. At low temperatures, the nitrate ions can orient with the electric field in the PVDF, perhaps accounting for some of the peak shifts and linewidth changes in the 804 cm^−1^ peak in the IR spectrum (relative to KNO_3_). As the materials are heated, an occluded solvent (DMF, H_2_O) is released. Concurrently, some of the nitrate begins to decompose to generate metal hydroxides. There also can be sufficient mobility for some aggregation to occur, so that some evidence of crystalline metal hydroxide or metal nitrate hydroxide nitrate double salts is observed. At a higher temperature, the nitrate decomposition accelerates and metal hydroxides dehydrate to give the final metal oxide product. The PVDF catalyzes the nitrate decomposition by polarizing the N–O bonds in the nitrate ion, allowing the bond cleavage to occur at lower temperatures. In the cases of the 2+ metal ion, a simple migration of a single oxide ion to a single metal ion allows for relatively rapid metal oxide formation. In the case of the 3+ metal ion, the migration events are slower because multiple ions need to aggregate to form the product. Thus, the catalysis induced by the PVDF is negated by the slow step of the aggregation of multiple metal and oxide ions.

## 4. Conclusions

In this study, we demonstrate the catalytic behavior of PVDF on chemical reactions for the first time. The temperature required to complete the thermal decomposition of 2+ charged metal nitrates shifted to a lower value when it is in PVDF thin films than the pure metal nitrate hydrates. In contrast, when the metal ion was 3+, the temperature shift was much smaller or to a higher temperature. The chemical reaction products were identified by XRD and gas phase and solid phase FTIR spectroscopy. The data for the intermediates was consistent with the formation of hydroxides while the final decomposition products were all oxides. The gas phase products identified were predominantly NO_2_ and H_2_O, for both pure metal nitrate hydrates or PVDF-doped materials. In cases where the product oxides contained a 3+ metal ion, the amount of NO_2_ detected was significantly smaller. This indicated that these oxides catalyzed the decomposition of NO_2_, presumably to N_2_ and O_2_. 

This catalytic activity invoked by the PVDF is hypothesized to result from the internal electric field. When PVDF is doped with metal nitrate hydrates, it significantly enhances the ferroelectric β-phase. The internal electric field orients the nitrate ions and polarizes the N-O bond. This promotes bond-cleavage, both heterolytically and homolytically. The formation of metal oxides is relatively fast for the 2+ metal ions since only two ions are required to encounter each other. In contrast, when the metal ion is in the 3+ state, multiple anions and cations are required to aggregate for the oxide product. In this case, while the PVDF still may promote decomposition of the nitrate ion, the formation of the oxide in the solid phase becomes the rate limiting step.

## Figures and Tables

**Table 1:** Composition parameters for PVDF samples.

Sample	p in M(NO_3_)_x_·pH_2_O (±0.1)	Mole Fraction of M in Casting Solution (±0.05)	Mole Fraction of M in Films (±0.05)	β-Phase Fraction, FTIR (±0.08)	β-Phase Fraction, XRD (±0.04)	ΔT, Oxide Onset T in M^n+^/PVDF (°C, ±5°)
Zn^2+^/PVDF	5.8	4.05	4.80	0.70	0.31	−114
Cu^2+^/PVDF	2.8	3.91	4.90	0.67	0.48	−24
Ni^2+^/PVDF	5.3	4.17	3.44	0.67	0.23	−170
Co^2+^/PVDF	5.7	4.07	4.60	0.69	0.32	−157
Fe^3+^/PVDF	8.4	4.14	3.21	0.69	0.30	+66
Cr^3+^/PVDF	8.2	4.14	4.44	0.69	0.47	−16
PVDF				0.64	0.16	

## Data Availability

All data used is contained in the paper.

## Supplementary Materials

The following are available online at https://www.mdpi.com/article/10.3390/polym13183112/s1, Figure S1: Room Temperature IR spectra showing deconvolution in the 730–850 cm^−1^ region; Figure S2: XRD for PVDF and doped PVDF; Figure S3: Gas phase IR spectra of thermally decomposed metal nitrate hydrates; Figure S4: XRD of the oxide metal nitrate hydrate thermal decomposition products; Figure S5: XRD of intermediates of the metal nitrate hydrate thermal reactions; Figure S6: XRD of intermediates of doped PVDF thermal reactions; Figure S7: XRD of final products of doped PVDF thermal reactions; Figure S8: FTIR of intermediates of doped PVDF thermal reactions; Figure S9: FTIR of final products of doped PVDF thermal reactions; Table S1: Deconvolution of the IR spectra results for unheated samples.
