# Supplementary material for: Catalysis of the Thermal Decomposition of Transition Metal Nitrate Hydrates by Poly(vinylidene difluoride)
Source: Polymers (Basel). 2021 Sep 15;13(18):3112. doi: 10.3390/polym13183112 (PMC8467513; doi:10.3390/polym13183112)
Supplement: Supplementary file 1 [file polymers-13-03112-s001.zip › polymers-1378675-supplementary.pdf]

Catalysis of the Thermal Decomposition of Transition Metal Nitrate Hydrates by Poly(vinylidene difluoride)

Lasanthi Sumathirathne, William B. Euler*
Department of Chemistry
University of Rhode Island
140 Flagg Road
Kingston, RI 02881

Supporting Information

Table of Contents

Figure S1: Room Temperature IR spectra showing deconvolution in the 730 – 850 cm^{-1} region 2

Table S1: Deconvolution of the IR spectra results for unheated samples 3

Figure S2: XRD for PVDF and doped PVDF 4

Figure S3: Gas phase IR spectra of thermally decomposed metal nitrate hydrates 5

Figure S4: XRD of the oxide metal nitrate hydrate thermal decomposition products 6

Figure S5: XRD of intermediates of the metal nitrate hydrate thermal reactions 7

Figure S6: XRD of intermediates of doped PVDF thermal reactions 8

Figure S7: XRD of final products of doped PVDF thermal reactions 9

Figure S8: FTIR of intermediates of doped PVDF thermal reactions 10

Figure S9: FTIR of final products of doped PVDF thermal reactions 11

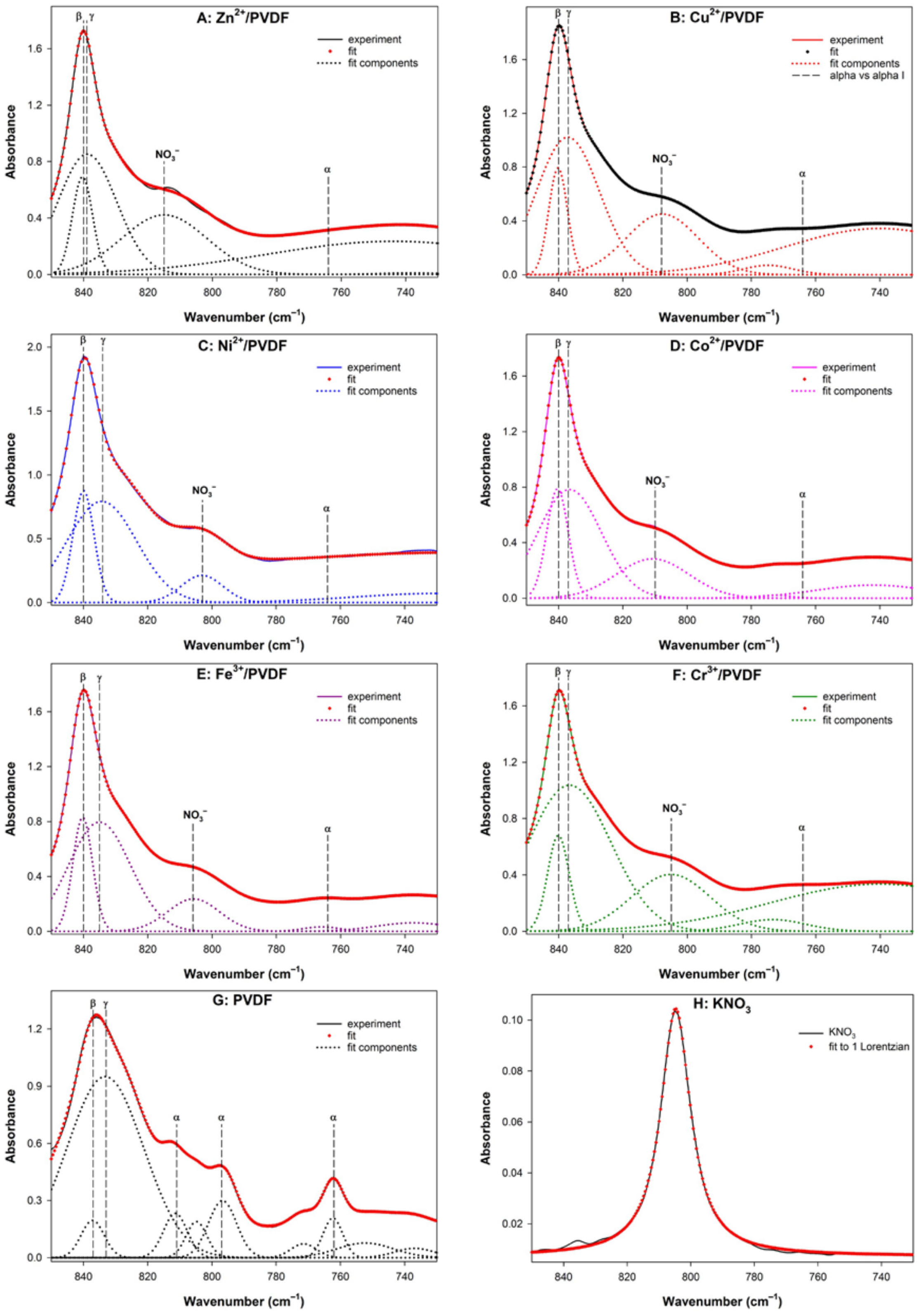

Figure S1. Deconvoluted IR data of room temperature PVDF-doped/ undoped thin films at 850-720 cm^{-1} region to identify the effect from nitrate peak. **A** Zn^{2+}/ PVDF; **B** Cu^{2+}/ PVDF; **C** Ni^{2+}/ PVDF; **D** Co^{2+}/ PVDF; **E** Fe^{3+}/ PVDF; **F** Cr^{3+}/ PVDF; **G** PVDF; **H** KNO_3. The α-, β-, γ-phase, and NO_3^- marker peaks are labeled.

Table S1. Peak maxima and linewidths found from deconvolution of IR spectra at room temperature. The spectra are fit to Gaussian functions with the reported linewidths being the full width at half maximum. The uncertainties on all of the fits are ±1 cm^{-1} or less.

Sample	C=O region Peak 1			C=O region Peak 2			NO_3^- region	
	Peak maximum (cm^{-1})	Linewidth (cm^{-1})	Relative Intensity	Peak maximum (cm^{-1})	Linewidth (cm^{-1})	Relative Intensity	Peak maximum (cm^{-1})	Linewidth (cm^{-1})
Zn^{2+}/PVDF	1639	28	0.21	1657	12	0.80	815	16
Cu^{2+}/PVDF	1635	30	0.47	1656	11	0.58	808	13
Ni^{2+}/PVDF	1633	38	0.64	1655	10	0.39	803	8
Co^{2+}/PVDF	1642	41	0.29	1657	11	0.60	811	13
Fe^{3+}/PVDF	1643	37	0.17	1653	11	0.83	806	9
Cr^{3+}/PVDF	1642	21	0.95				805	14
PVDF								
KNO_3							806	7
DMF				1660	17			

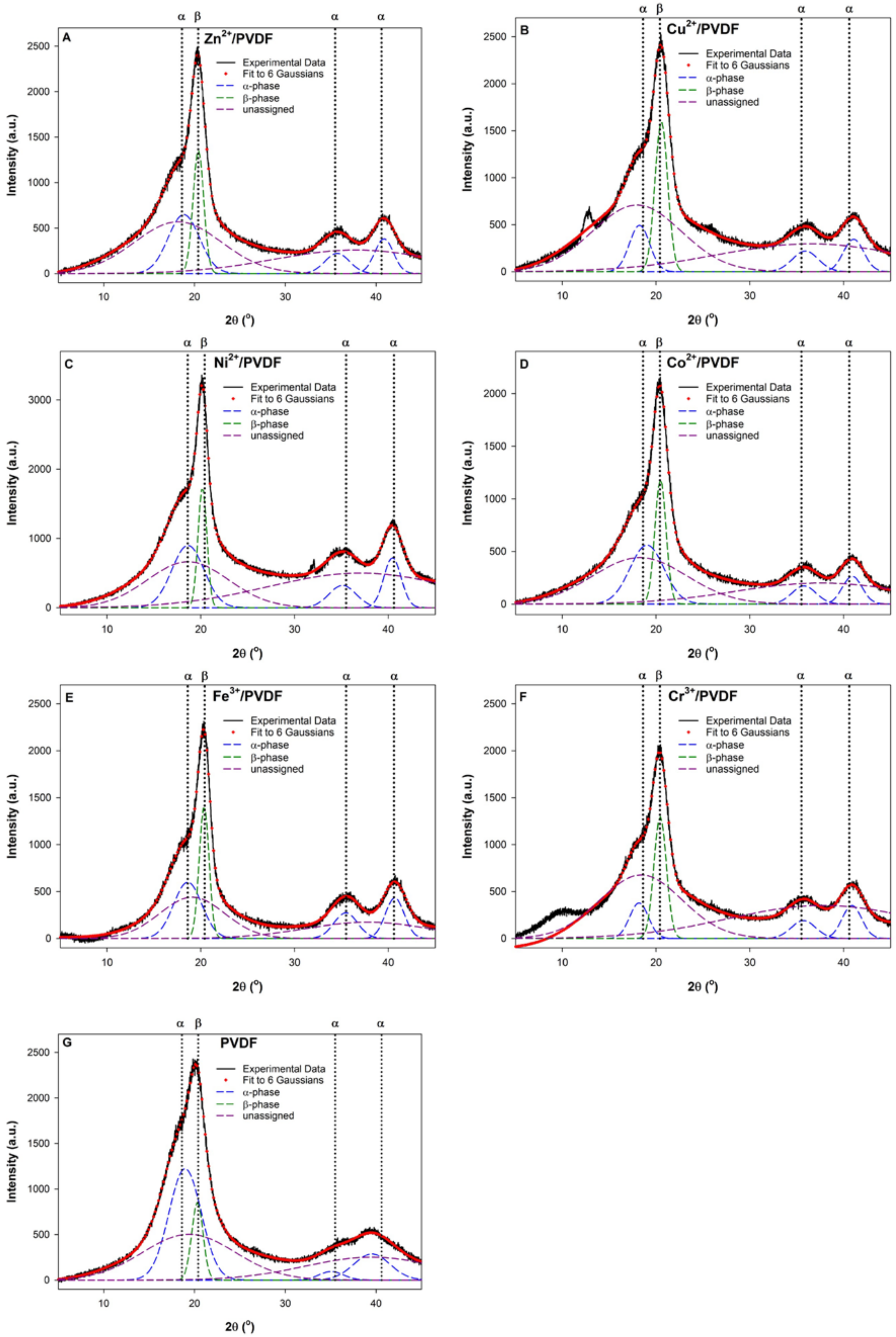

Figure S2. XRD data for doped/undoped PVDF films showing the deconvolutions. **A**: Zn^{2+}/PVDF. **B**: Cu^{2+}/PVDF. **C**: Ni^{2+}/PVDF. **D**: Co^{2+}/PVDF. **E**: Fe^{3+}/PVDF. **F**: Cr^{3+}/PVDF. **G**: PVDF.

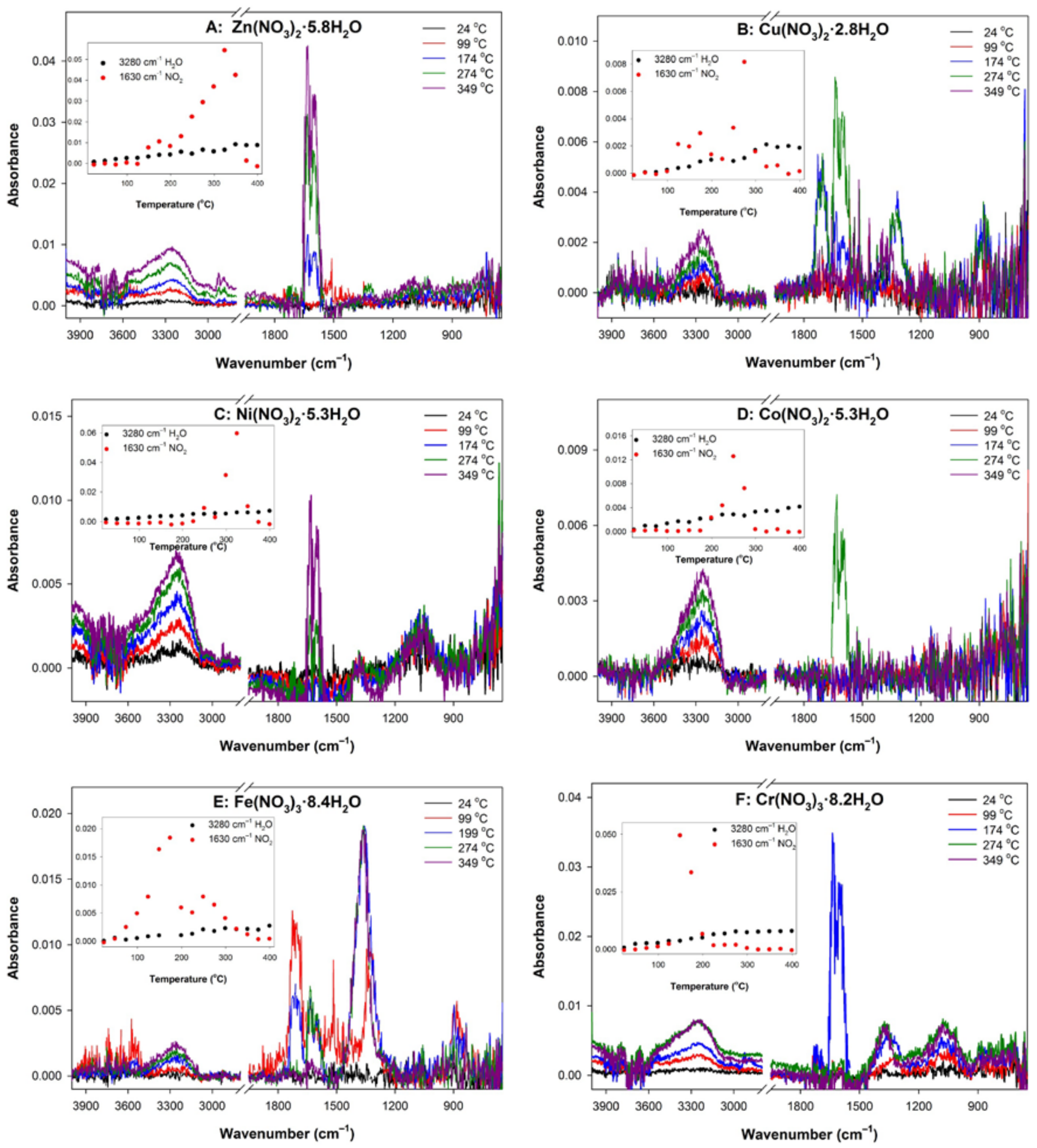

Figure S3. Gas phase IR spectra of the volatile products of the thermal decomposition of pure metal nitrate hydrates as a function of temperature. **A** $Zn(NO_3)_2 \cdot 5.8H_2O$; **B** $Cu(NO_3)_2 \cdot 2.8H_2O$; **C** $Ni(NO_3)_2 \cdot 5.3H_2O$; **D** $Co(NO_3)_2 \cdot 5.3H_2O$; **E** $Fe(NO_3)_3 \cdot 8.4H_2O$; **F** $Cr(NO_3)_3 \cdot 8.2H_2O$. The insets show the temperature dependence of the H_2O (3280 cm^{-1}) and NO_2 (1630 cm^{-1}) peak intensities.

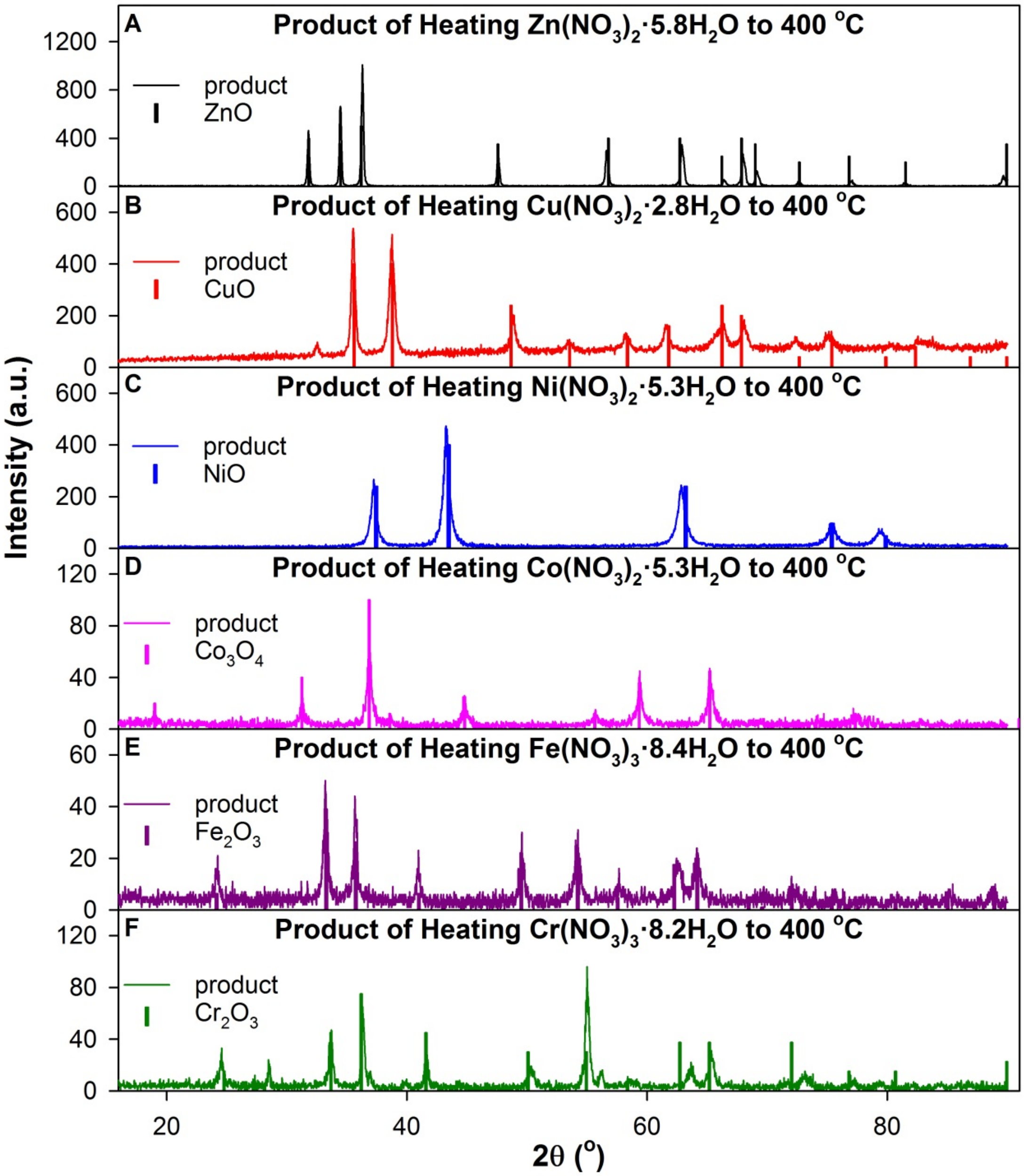

Figure S4. XRD results of metal nitrate hydrates heated to 400 ^{o}C. **A**: $Zn(NO_3)_2{\cdot}5.8H_2O$; **B**: $Cu(NO_3)_2{\cdot}2.8H_2O$; **C**: $Ni(NO_3)_2{\cdot}5.3H_2O$; **D**: $Co(NO_3)_2{\cdot}5.3H_2O$; **E**: $Fe(NO_3)_3{\cdot}8.4H_2O$; **F**: $Cr(NO_3)_3{\cdot}8.2H_2O$. The vertical bars show the literature reference values for the indicated oxides. The data for the oxides was taken from International Center for Diffraction Data (ICDD) reference library.

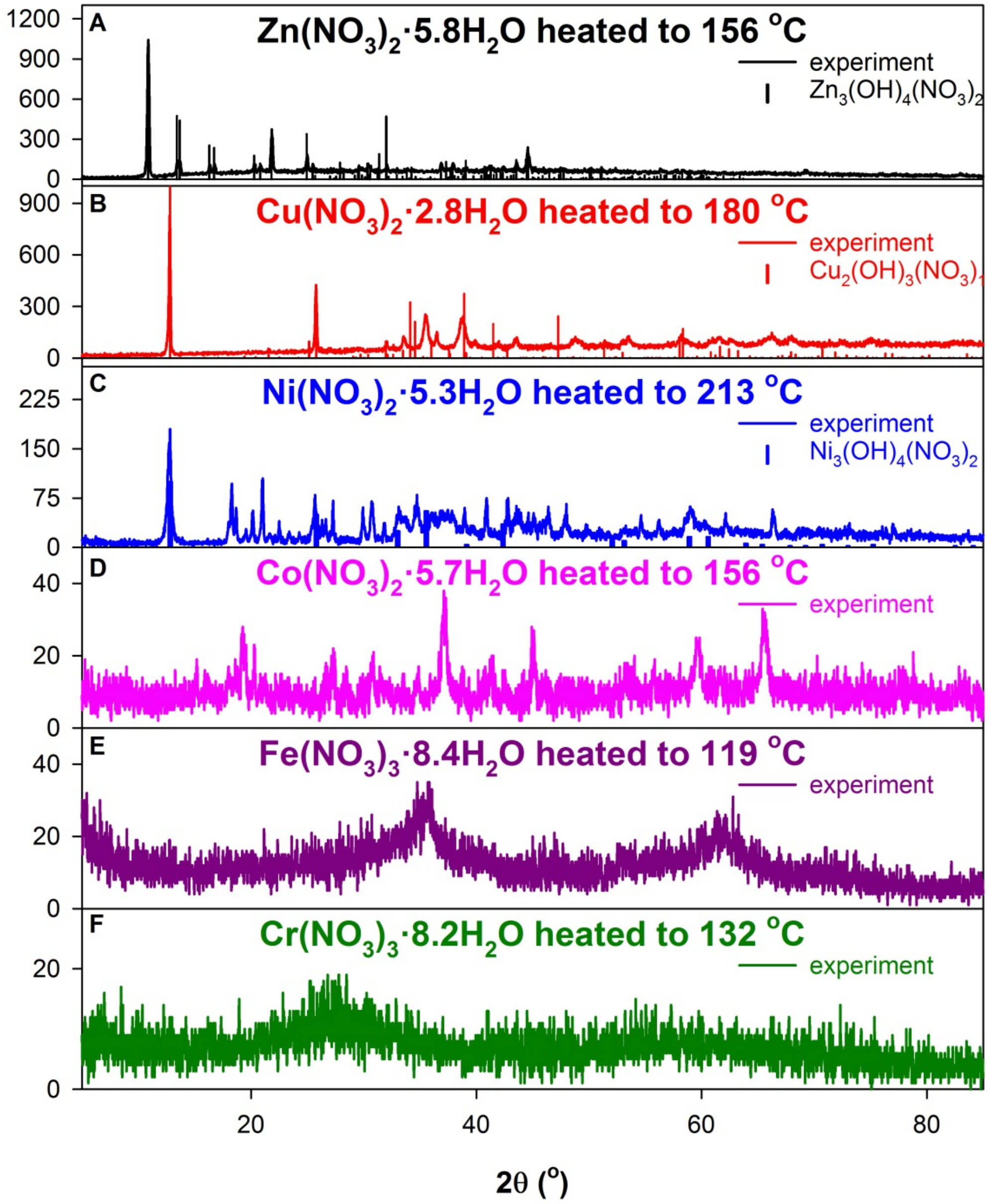

Figure S5. XRD results of metal nitrate hydrates heated to the onset temperature of the intermediate product. **A**: $Zn(NO_3)_2{\cdot}5.8H_2O$ heated to 156 °C; **B**: $Cu(NO_3)_2{\cdot}2.8H_2O$ heated to 180 °C; **C**: $Ni(NO_3)_2{\cdot}5.3H_2O$ heated to 213 °C; **D**: $Co(NO_3)_2{\cdot}5.3H_2O$ heated to 156 °C; **E**: $Fe(NO_3)_3{\cdot}8.4H_2O$ heated to 119 °C; **F**: $Cr(NO_3)_3{\cdot}8.2H_2O$ heated to 132 °C. The vertical bars show the literature reference values for the indicated hydroxide/nitrate double salts. The data for the double salts was taken from International Center for Diffraction Data (ICDD) reference library.

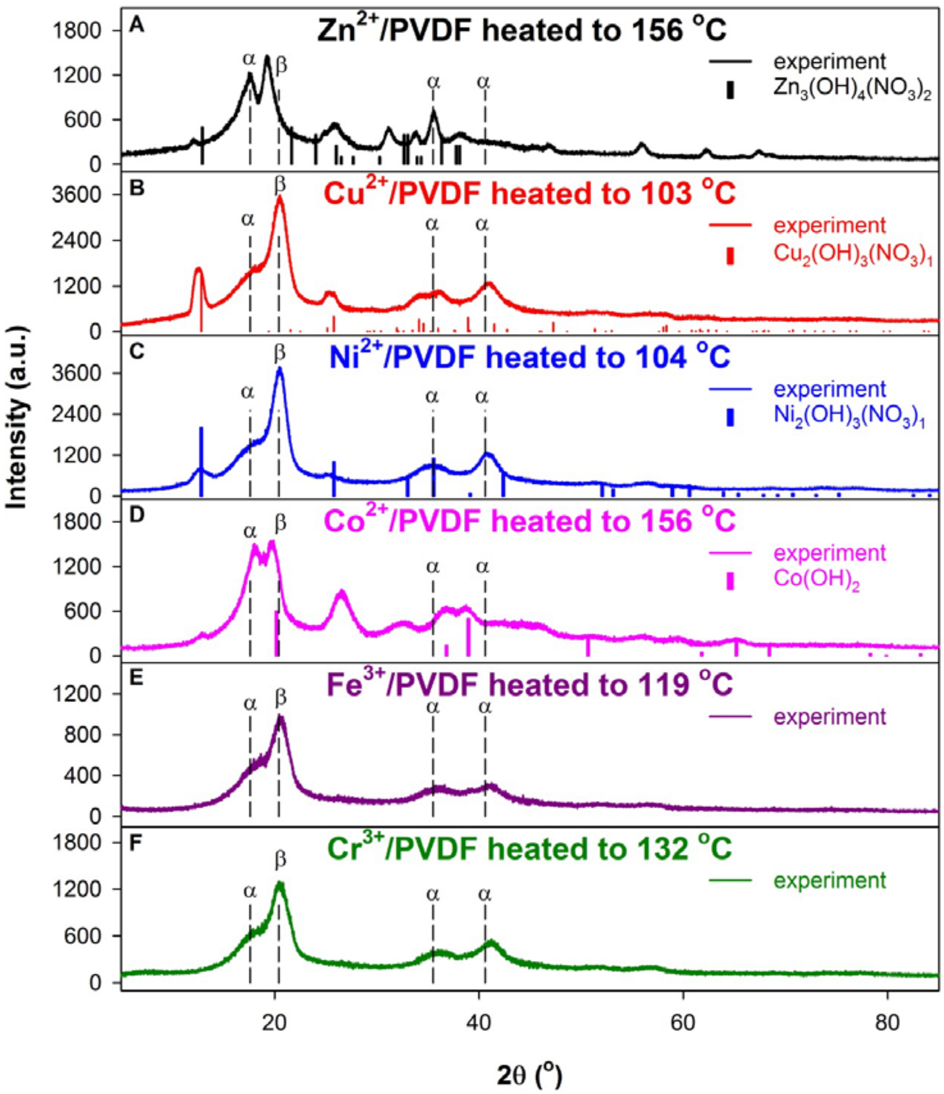

Figure S6. XRD results of doped PVDF heated to the onset temperature of the intermediate product. **A**: Zn^{2+}/PVDF heated to 156 °C; **B**: Cu^{2+}/PVDF heated to 103 °C; **C**: Ni^{2+}/PVDF heated to 104 °C; **D**: Co^{2+}/PVDF heated to 156 °C; **E**: Fe^{3+}/PVDF heated to 119 °C; **F**: Cr^{3+}/PVDF heated to 132 °C. The vertical bars show the literature reference values for the indicated hydroxide/nitrate salts. The data for the salts was taken from International Center for Diffraction Data (ICDD) reference library.

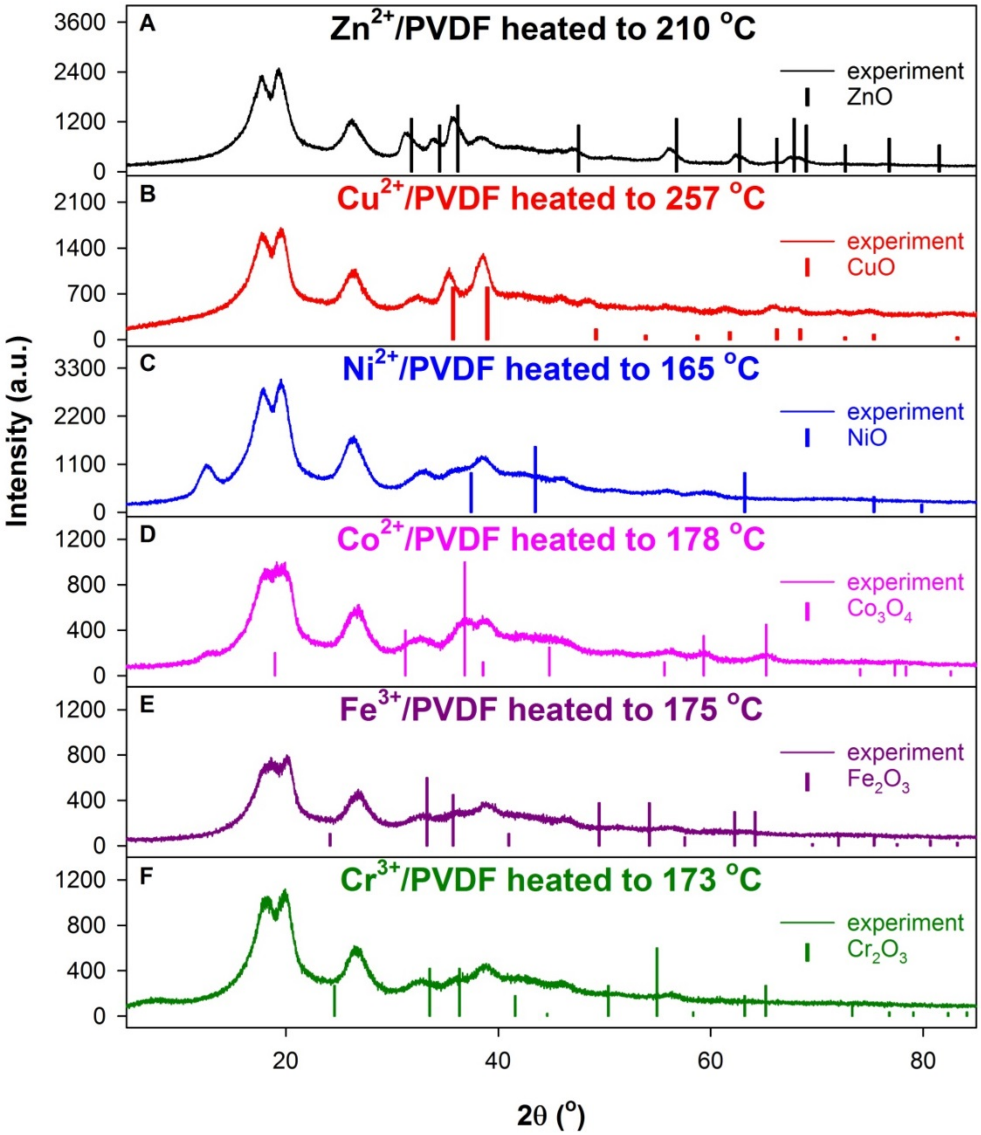

Figure S7. XRD results of doped PVDF heated to the onset temperature of the final product. **A**: Zn^{2+}/PVDF heated to 210 °C; **B**: Cu^{2+}/PVDF heated to 257 °C; **C**: Ni^{2+}/PVDF heated to 165 °C; **D**: Co^{2+}/PVDF heated to 178 °C; **E**: Fe^{3+}/PVDF heated to 175 °C; **F**: Cr^{3+}/PVDF heated to 173 °C. The vertical bars show the literature reference values for the indicated oxides. The data for the oxides was taken from International Center for Diffraction Data (ICDD) reference library.

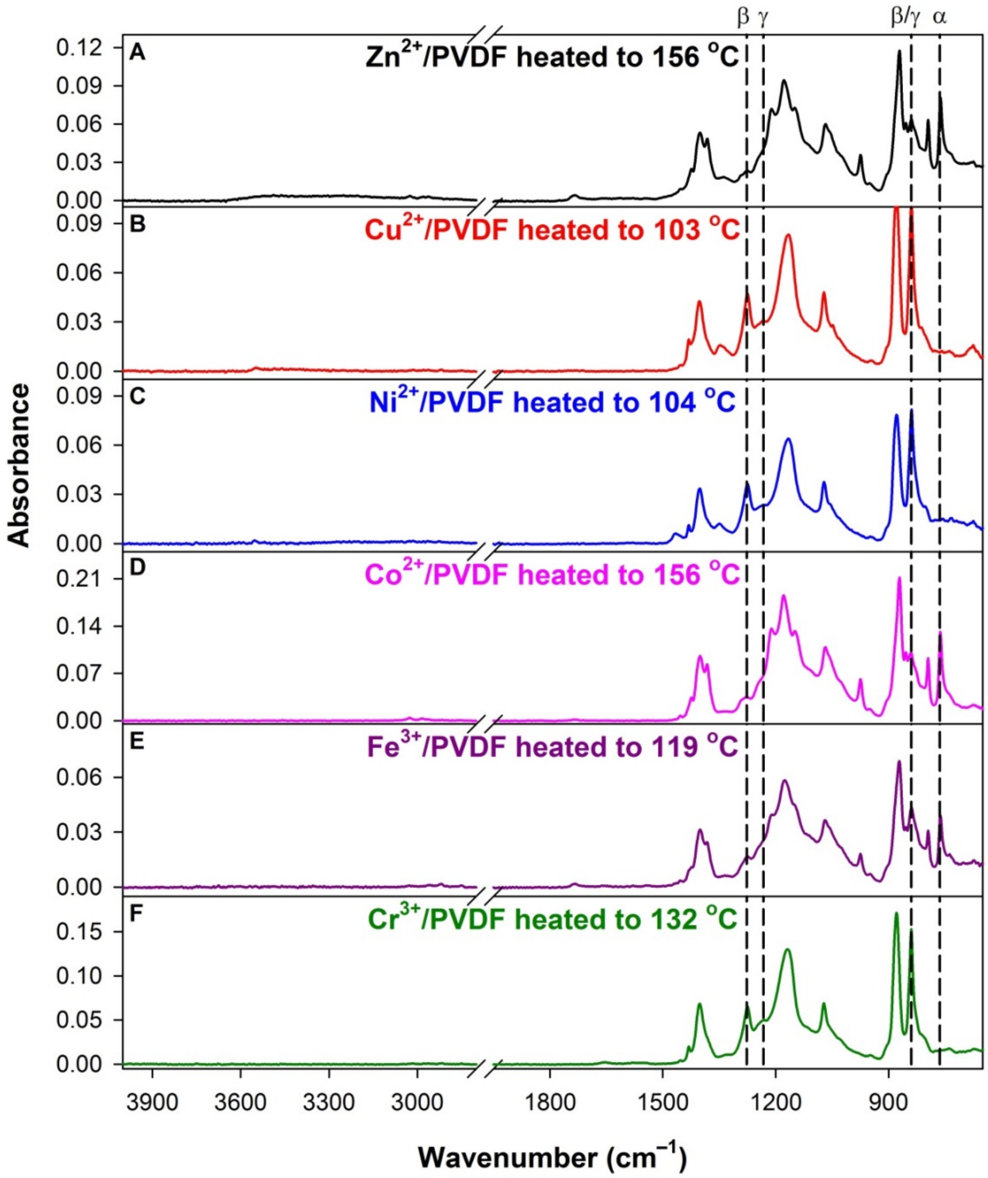

Figure S8. FTIR results of doped PVDF heated to the onset temperature of the intermediate product. **A**: Zn^{2+}/PVDF heated to 156 ^{o}C; **B**: Cu^{2+}/PVDF heated to 103 ^{o}C; **C**: Ni^{2+}/PVDF heated to 104 ^{o}C; **D**: Co^{2+}/PVDF heated to 156 ^{o}C; **E**: Fe^{3+}/PVDF heated to 119 ^{o}C; **F**: Cr^{3+}/PVDF heated to 132 ^{o}C.

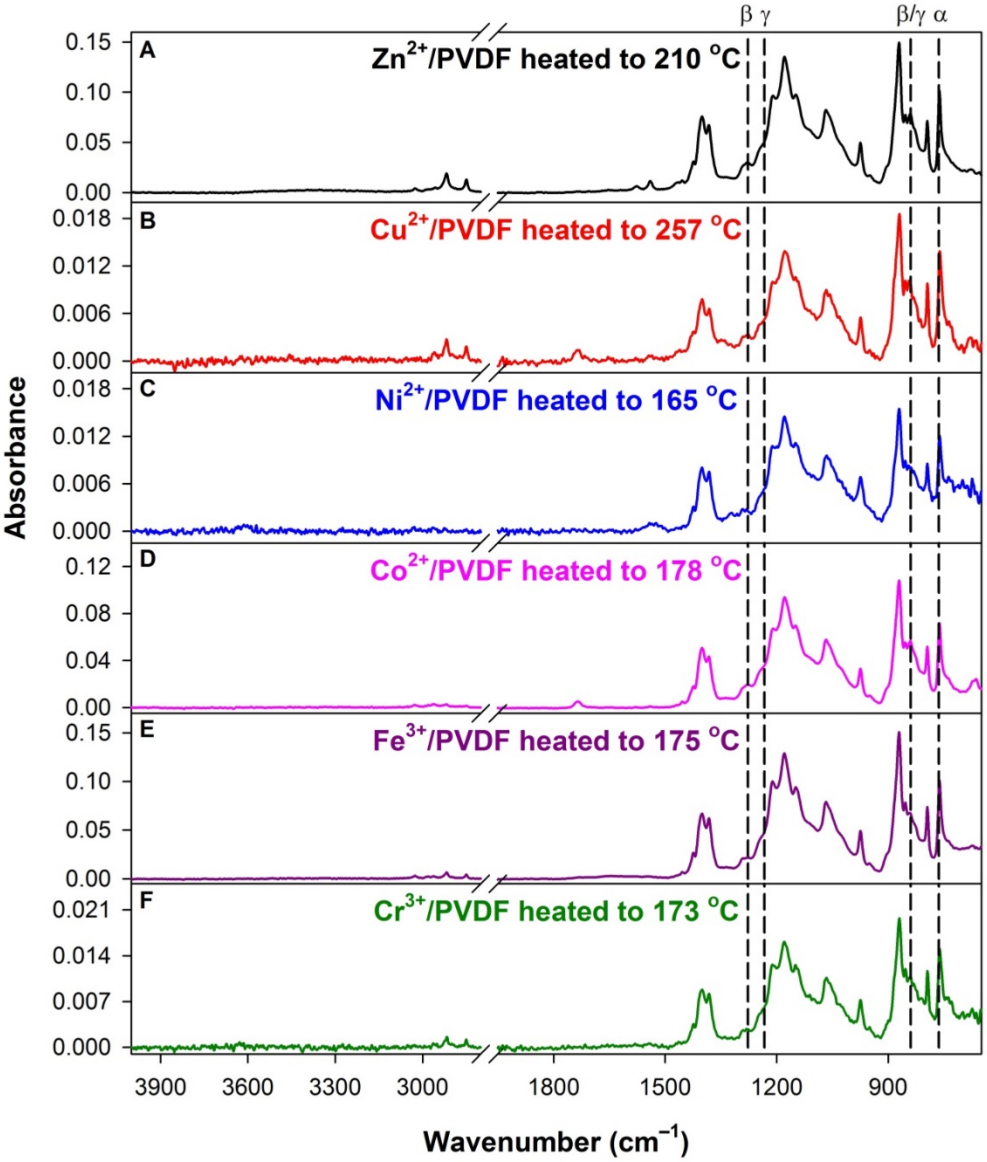

Figure S9. FTIR results of doped PVDF heated to the onset temperature of the final product. **A**: Zn^{2+}/PVDF heated to 210 °C; **B**: Cu^{2+}/PVDF heated to 257 °C; **C**: Ni^{2+}/PVDF heated to 165 °C; **D**: Co^{2+}/PVDF heated to 178 °C; **E**: Fe^{3+}/PVDF heated to 175 °C; **F**: Cr^{3+}/PVDF heated to 173 °C.
